# The significance of biological samples from pregnant women in cervical intraepithelial neoplasia

**DOI:** 10.3389/fmed.2025.1645567

**Published:** 2025-09-16

**Authors:** Xue Mi, Maharjan Rashmi, Zangyu Pan, Di Wu, Jinwei Miao

**Affiliations:** Beijing Obstetrics and Gynecology Hospital, Capital Medical University, Beijing Maternal and Child Health Care Hospital, Beijing, China

**Keywords:** cervical intraepithelial neoplasia, cervical cytology, human papilloma virus, pregnancy, colposcopy

## Abstract

**Background and aims:**

Cervical cancer remains a significant threat to women’s health, with pregnant women representing a particularly vulnerable population. This study aimed to investigate the impact of cervical intraepithelial neoplasia (CIN) on pregnancy outcomes using longitudinal biological sample analysis.

**Methods:**

We conducted a retrospective study of 125 pregnant women who underwent vaginal examination following abnormal cervical cytology and/or positive human papillomavirus (HPV) testing. Suspected cases underwent colposcopy-directed cervical biopsy performed by experienced clinicians (10 year of work experience) during pregnancy. Postpartum follow-up included repeat cervical cytology, HPV testing, and colposcopic biopsy when indicated.

**Results:**

Among the 125 patients, 34 underwent colposcopic biopsy during pregnancy, with histopathological results demonstrating strong concordance with colposcopic findings (kappa = 0.82, *p < 0.001). Postpartum follow-up within one year of delivery included colposcopy and cervical biopsy in 98 patients. Multivariate logistic regression analysis revealed that persistent cervical cytological abnormalities (OR 9.838; 95% CI 3.851–25.135; *p < 0.001) were significantly associated with abnormal colposcopic findings.

**Conclusion:**

For pregnant women declining cervical biopsy during pregnancy, colposcopy represents a safe and clinically valuable diagnostic tool. Persistent cervical cytological abnormalities, but not HPV positivity, were identified as a significant risk factor for CIN2 + persistence.

## Introduction

Cervical carcinoma represents the fourth most prevalent gynecologic malignancy worldwide (1) and ranks as the fourth leading cause of cancer-related mortality among women globally (2, 3). The disease spectrum includes precancerous lesions such as cervical intraepithelial neoplasia grade 2 or higher (CIN2+) and carcinoma *in situ* (CIS), which may persist for extended periods. These lesions demonstrate variable clinical behavior, with potential for spontaneous regression, persistence, or progression to invasive carcinoma. The incidence of cervical cancer during pregnancy ranges from 0.8 to 1.5 cases per 10,000 births (4, 5), with rising maternal age contributing to an increased prevalence of both cervical cancer and CIN in this population (6). Currently, evidence CIN during pregnancy remains limited, and optimal clinical management remains a subject of ongoing debate.

Recent advances in molecular oncology have significantly enhanced our understanding of cervical carcinogenesis, with numerous studies investigating tumor biology and morphology (7–9). While novel biomarkers and targeted therapeutic agents are under development for various stages of cervical cancer, human papillomavirus (HPV) testing and cervical cytology remain the cornerstone of clinical practice for detecting CIN in pregnant women. Pregnancy is a vulnerable phase where women are immunosuppressive and undergo a variety of hormonal changes, which may lead to more chances of acquiring HPV infection (10). Precursor lesions and uterine cervical cancer are caused by various oncogenic types of HPV, with a higher prevalence of types 16 and 18, which are subjected to cause up to 70% of cervical cancer precursor lesions (11). About 25% of the reproductive age group especially 20 to 30 year young women are especially infected by HPV (12). During pregnancy cervical changes can occur, even though pregnancy itself is not a risk factor for worsening cervical lesions, such as changes in the shape or size of the cervix, changes in squamous and glandular epithelial cells and increased vascularity can impede the interpretation of cervical cytology and colposcopy (13). Pregnant patients of 21 years and older are screened during the first trimester and their abnormal cytology is managed according to the guidelines for the general population. In the case of CIN diagnosed during pregnancy, management strategies should be considered depending on the size of the tumour, the diagnostic image findings, gestational age at the time of diagnosis, and the desire of the patient to continue the pregnancy (14).

Pregnancy-associated CIN represents a unique clinical entity with distinct diagnostic and management challenges. Current evidence remains limited and often contradictory regarding the natural history of CIN during pregnancy. Our study specifically examines this high-risk population to evaluate: (1) the predictive value of cervical cytology and HPV testing for disease progression, and (2) the clinical outcomes of pregnancy-complicated CIN, with particular emphasis on developing optimized surveillance strategies.

## Materials and methods

This was a single-center retrospective study. This retrospective cohort study included 125 pregnant patients who underwent concurrent cervical cytology, HPV testing, and colposcopic evaluation at Beijing Obstetrics and Gynecology Hospital, Capital Medical University (Beijing, China) between January 2015 and December 2019. Among these 125 pregnant women, all underwent colposcopy due to abnormal cervical cytology or high-risk HPV. The study protocol received ethical approval from the Institutional Review Board of Beijing Obstetrics and Gynecology Hospital, Capital Medical University (Beijing, China).

Inclusion criteria: 1. Pregnant women aged 21–45 years; 2. Cytological abnormalities (ASC-US or greater) and/or positive high-risk HPV test during pregnancy; 3. Completion of colposcopic examination during gestation. Exclusion criteria: 1. Current or history of significant pregnancy complications (including but not limited to placenta previa or active vaginal bleeding); 2. Known HIV infection or immunocompromised status (primary or secondary); 3. Diagnosis or treatment of cervical lesions within 12 months preceding conception; 4. Pregnancy termination prior to completion of diagnostic workup. The collected data include age, cervical cytology, HPV results, colposcopy results and cervical biopsy.

Classifications of cytology and histopathology refer to the cervical cancer screening guidelines (2020), which created by the American Cancer Society (ACS).

The colposcopy examination results of this study are defined as follows (15): high-grade squamous intraepithelial lesion (HSIL), cannot rule out high-grade squamous intraepithelial lesion (ASC-H) and invasive carcinoma were classified as “severe colposcopy impression,” while cervicitis, atypical squamous cells of undetermined significance (ASC-US), and low-grade squamous intraepithelial lesion (LSIL) were classified as “mild colposcopy impression”.

For the pathological results of cervical biopsy, in this study, cases with “mild colposcopy impression” and pathological results of CIN1 or cervicitis were defined as “colposcopy-histopathological consistency.” The cases with “severe colposcopy impression” and pathological results of CIN2-3 or invasive cancer examined were defined as “colposcopy-histopathological consistency.” Cases where the colposcopy results are worse than the histopathological diagnosis are defined as “overestimated colposcopy,” and cases where the colposcopy results were better than the histopathological diagnosis were defined as “underestimated colposcopy”.

Postpartum follow-ups of all patients were done within one year after delivery. Patients were interviewed through a call to collect adequate information. Final follow-up results were compared with initial results to determine cervical lesions outcomes such as progression, persistence, regression and complete regression in terms of cervical cytology, HPV, colposcopy and cervical biopsy. In addition, outcome of patients was also analyzed interms of parity, mode of delivery and area.

Statistical analysis was performed in SPSS (version 27.0). Clinical categorical variables were presented in numbers and percentages. Logistic regression was applied to find the predictive role of disease outcome. The consistency test of diagnostic tests was conducted using Cohen’s Kappa test. Associations were shown as odds ratios (OR) with 95% confidence intervals (CI), and a *p* < 0.05 was considered statistically significant.

## Result

### Clinical characteristics of pregnant women

A total of 125 pregnant women were enrolled in this study, all of whom underwent colposcopy due to abnormal results of cervical cytology or HPV tests. The age of the enrolled patients was 31.0 ± 3.8 years (the youngest was 23 years and the oldest was 43 years), and other conditions were as follows (see Table 1).

**Table 1:** General characteristics of patients.

	Number (%)
Cervical cytology	
ASCUS	24 (19.2)
LSIL	49 (39.2)
ASC-H	7 (5.6)
HSIL	45 (36.0)
HPV	
Negative	17 (13.6)
Positive	108 (86.4)
Delivery mode	
Cesarean section	57 (45.6)
Vaginal delivery	68 (54.4)
Previous childbirth history	
0	83 (66.4)
1	40 (32.0)
2	2 (1.6)

### Colposcopy examination results

26 of 125 patients underwent colposcopy examinations twice: the first examination is in the early stage of pregnancy, and the second examination is in the middle stage of pregnancy. In the 26 cases, 22 cases were HSIL twice; 2 cases were HSIL in the first test and early invasion carcinoma in the second test; 1 case was LSIL in the first test and the second result was HSIL; 1 case was LSIL obtained in both colposcopy tests; Except for two cancer patients who underwent active surgery in the third trimester of pregnancy, the other 24 patients did not receive treatment during pregnancy. Among 24 cases, 7 were persistent (29.2%) and 17 regression (70.8%) including 9 complete regression cases (see Table 2).

**Table 2:** Colposcopic impression and cervical biopsy.

	Number (%)
Colposcopic impression	
Cervicitis	16 (12.8)
LSIL	37 (29.6)
HSIL	67 (53.6)
Cancer	5 (4.0)
Cervical biopsy	
CIN1	7 (20.6)
CIN2+	22 (64.7)
Cancer	5 (14.7)

The results of colposcopy during pregnancy showed that there were 72 cases (57.6%) of “severe colposcopy impression,” including 67 cases of HSIL and 5 cases of invasive cervical cancer, and 53 cases (42.4%) of “mild colposcopy impression,” including 16 cases of cervicitis. There were 37 cases of LSIL. Only 34 patients with “severe colposcopy impression” underwent colposcopy cervical tissue biopsy. The pathological results were cervicitis 2(5.9%), CINI 5 (14.7%), CINII 7 (20.6%), CINIII 15 (44.1%), and cancer 5(14.7%). “Colposcope and histopathological concordance” 32 cases (94.1%), “colposcope examination overestimation” in 2 cases (5.9%), “colposcope examination underestimation” 0 cases (0%) (see Table 3).

**Table 3:** Colposcopic impression accuracy.

	Number (%)	K* coefficient	95%CI	Standard error	*p*-value
Concordance	32 (94.1%)	0.882	0.699–1.043	0.081	<0.001
Colposcopy underestimation	0 (0%)				
Colposcopy overestimation	2 (5.9%)				

During the postpartum follow-up, among the 125 patients, 5 patients were diagnosed with cervical cancer during pregnancy and received timely treatment, surviving until now. The clinical data of their postpartum re-examination were no longer included, and the follow-up of another 4 patients refused. Ultimately, 116 patients underwent cervical cytology and HPV tests within 6 weeks to 10 months after delivery. The results of cervical cytology examination are as follows: HSIL 31 (26.7%), LSIL 35 (30.2%), ASCUS 12 (10.3%), ASC-H 1 (0.9%), NILM 37 (31.9%). There were 76 cases (65.5%) positive for HPV testing after childbirth and 40 cases (34.5%) negative.

Among the 116 patients, 98 patients underwent colposcopy and cervical tissue biopsy. As the results of cervical cytology and HPV were negative, colposcopy was not performed in 18 cases, and it was considered to have completely regressed. Colposcopy was used as a comparison of outcomes during pregnancy and postpartum. Among the patients with colposcopy results of LSIL during pregnancy, 13 cases (35.1%) were persistent, 7 cases (18.9%) were progressive, and 17 cases (46.0%) were regression. Among the patients with HSIL in the colposcopy examination results during pregnancy, 29 cases (47.5%) were persistent, 0 cases (0.0%) progressed, 32 cases (52.5%) regressed, and 20 cases (32.8%) completely regressed (see Table 4).

**Table 4:** Comparison of colposcopy impression during pregnancy and postpartum.

Colposcopy impression	LSIL	HSIL	X^2^	*P*
	Number (%)	Number (%)		
Persistence/progression	20 (54.0%)	29 (47.5%)	0.391	0.532
Regression	17 (46.0%)	32 (52.5%)		

The influencing factors of the outcome of cervical intraepithelial neoplasia were analyzed by multiple logistic regression. The outcome was based on the results of colposcopy. Due to the negative results of cervical cytology and HPV, cases without colposcopy are considered to have complete regression of cervical intraepithelial neoplasia. Persistent abnormal cytological results are significantly associated with the outcome of CIN. High-risk HPV infection, age, parity, mode of delivery and region were not related to the outcome of CIN (see Table 5).

**Table 5:** Analysis of outcome factors of cervical intraepithelial neoplasia.

Category		Persistence/progression	Regression	Univariate		Multivariate	
		Number (%)	Number (%)	Odds ratio (95% CI)	*P*-value	Odds ratio (95% CI)	*P*-value
Age (years)	<35	46 (48.0)	50 (52.0)				
	≥35	9 (45.0)	11 (55.0)	1.124 (0.427–2.959)	0.812	1.446 (0.437–4.783)	0.545
Cervical cytology	Yes^a^	45 (81.8)	10 (18.2)				
	No^a^	19 (31.1)	42 (68.9)	9.947 (4.153–23.829)	<0.001	9.838 (3.851–25.135)	<0.001
High-risk HPV	Yes^b^	39 (70.9)	16 (29.1)				
	No^b^	30 (49.2)	31 (50.8)	2.519 (1.168–5.432)	0.018	1.451 (0.580–3.629)	0.426
Parity	0	42 (53.8)	36 (46.2)				
	≥1	19 (50.0)	19 (50.0)	0.857 (0.394–1.863)	0.697	0.975 (0.384–2.475)	0.957
Delivery mode	Vaginal	29 (43.3)	38 (56.7)				
	CS	26 (53.1)	23 (46.9)	1.481 (0.706–3.106)	0.298	1.732 (0.710–4.221)	0.227
Area	beijing	29 (51.8)	27 (48.2)				
	Non-local	26 (43.3)	34 (56.7)	1.405 (0.676–2.920)	0.363	2.081 (0.826–5.243)	0.120

a Cervical cytology: No-regression. Yes-persistence/progression. Regression included 2 parts. One part: after re-examination, HISL or ASC-H was changed to LISL, ASCUS. Another part: after re-examination, NILM and LISL or ASCUS was changed to NILM. Persistence/progression included 3 parts. One part: after re-examination, HISL or ASC-H was changed to cervical cancer. Another part: after re-examination, LISL or ASCUS was changed to HISL, ASC-H or cervical cancer.

b High-risk HPV: No-regression, Yes-persistence. Regression means that after re-examination, high-risk HPV was changed to low-risk HPV or negative; persistence means that after re-examination, high-risk HPV was stable.

The biopsy results of cervical tissues in 98 patients were as follows: 29 cases (29.6%) of cervicitis, 29 cases (29.6%) of CIN1, 20 cases (20.4%) of CIN2, and 20 cases (20.4%) of CIN3. There were no new cases of cervical cancer among the patients during the postpartum follow-up. The biopsy rate of cervical tissue in pregnant women who underwent colposcopy during pregnancy was 27.2%, and that in pregnant women who underwent colposcopy after childbirth was 100.0%.

## Discussion

This study demonstrated a significantly higher rate of spontaneous regression of cervical lesions following delivery. Notably, we observed a remarkably low incidence of disease progression, with no cases progressing to invasive carcinoma during postpartum follow-up. These findings align with the 2019 American Society for Colposcopy and Cervical Pathology (ASCCP) guidelines, which endorse conservative management for pregnant women diagnosed with CIN2 + lesions, provided invasive disease is excluded through thorough colposcopic evaluation and biopsy (16). Our results further support the recommendation to defer definitive treatment for CIN2 + until the postpartum period in such cases. For patients with biopsy-confirmed cervical cancer, clinical management should be individualized based on gestational age.

The prognosis of cervical intraepithelial neoplasia (CIN) during pregnancy varies across studies. Vlahos et al. (17) reported that among 78 pregnant women with CIN2+, 61.6% (48/78) regressed to CIN1 postpartum, while 38.4% (30/78) exhibited persistent disease, with no progression to invasive carcinoma. Similarly, Mailath-Pokorny et al. (18) observed a 56.9% regression rate and 3.9% progression, whereas Yost et al. (19) noted 69.3% regression, 26.8% persistence, and 3.9% progression postpartum. In our cohort, 59.1% (39/66) of cases regressed, including 31.8% (21/66) with complete resolution, while 40.9% (27/66) persisted and none progressed. In contrast, a study of 154 pregnant women with CIN3 reported persistence, regression, and progression rates of 76.1, 20, and 3.2%, respectively (20). A meta-analysis further indicated that 1% of high-grade CIN cases during pregnancy progressed to cervical cancer (21). Although 3.7% of invasive cervical cancers in our study were diagnosed during pregnancy, no cases of CIN2 + progressed to malignancy on antenatal biopsy. The mechanism underlying postpartum CIN regression remains debated. Some studies suggest that cervical trauma during vaginal delivery and subsequent repair may promote lesion regression (9), while others report no significant difference in regression rates between vaginal and cesarean deliveries (22). Our findings align with the latter, indicating no association between delivery mode and cervical lesion outcome.

The results of this study show that the colposcopy examination results have a high consistency with cervical tissue biopsy. In an observational study, it was found that 47 patients (68.1%) were generally “Colposcopy-histopathological consistent,” among which 12 patients (17.4%) had underestimated colposcopy and 10 patients (14.5%) had overestimated colposcopy (15). At the same time, colposcopy assessment was recommended in the first half of pregnancy. Similarly, Grimm et al. (23) also found that when comparing the vaginal examination results with the pathological results, the consistency of CINI and CINII-III lesions was 33 and 81.8%, respectively. The latest guidelines of the American Society for Colposcopy and Cervical Pathology (ASCCP) in 2019 state that when histological HSIL (CINII or CINIII) is detected during the first colposcopy of pregnancy, colposcopy and laboratory tests (cytology/age-based HPV) at 12 or 24 weeks should be preferred, and colposcopy can be terminated at 4 weeks postpartum. Repeated biopsies can be performed when suspected invasive cancer is found during colposcopy or when lesion progression is suspected. The colposcopy examination results were studied and examined by professional clinicians (10 year of work experience). The results confirmed the high consistency between colposcopy examination and cervical tissue biopsy. Considering the risks such as vaginal bleeding, infection and miscarriage that may occur in cervical tissue biopsy, this study suggests that even without cervical tissue biopsy, the results of colposcopy are still reliable, and colposcopy is recommended during pregnancy. Furthermore, among the 125 pregnant women included in the group, 5 of them had colposcopy results suggesting suspicious carcinoma *in situ* when cervical cytology did not suggest cancer, and were confirmed as cervical cancer by tissue biopsy, providing key evidence for the early diagnosis in clinical diagnosis and treatment.

In this study, the rate of cervical biopsy among pregnant women was relatively low. The possible reasons for the analysis are as follows: First, from the patients, because they refused to undergo cervical biopsy during pregnancy and were worried about the complications resulting from it. Secondly, it comes from doctors, as they tend to avoid any procedures that may lead to obstetric complications, fearing possible medical disputes. According to the data of this study, although the proportion of cervical biopsy during pregnancy was relatively low, it did not lead to adverse outcomes for patients and reduced medical costs. Therefore, this study considers that the low rate of cervical biopsy among pregnant women was acceptable. However, if cancer or deterioration of the lesion is suspected, it is recommended to repeat the colposcopy examination and perform a biopsy if necessary. If a cervical biopsy was not performed during pregnancy, it is also recommended to actively follow up after giving birth to confirm the diagnosis. It has been reported that selective treatments for high-grade lesions during pregnancy, such as cold knife conization (CKC) and loop electro-surgical excision procedure (LEEP), can lead to bleeding, miscarriage and premature birth. Robinson et al. reported a high incidence of complications. Among 20 women who received LEEP treatment in the second and third trimester of pregnancy, there were 3 cases of preterm birth, 3 cases of severe blood loss, and 1 case of intrauterine fetal death (15). Therefore, it is not recommended to treat HSIL (CIN II or CIN III) in prenatal. Therefore, the role of colposcopy during pregnancy is to rule out cervical cancer. Precancerous lesions of the cervix can be managed as expected, but treatment can be safely postponed until postpartum. Only the colposcopy impression of invasive cancer requires more in-depth assessment and decision-making to align with the patient’s values and expectations.

Most of the potential diagnostic and prognostic biomarkers of cervical tumor lesions are based on the molecular mechanisms related to HPV infection (24, 25). Persistent infection with certain HPV genotypes is the main cause of cervical cancer (26).

Although cervical cytology tests and HPV tests are the first steps in cervical cancer screening. However, this study suggests that for pregnant women with CIN during pregnancy, cervical cytological examination seems to have more clinical value than HPV examination. The data of this study indicate that, compared with the postpartum data, the persistent abnormalities in cervical cytology during pregnancy are closely related to the persistence or progression of cervical lesions. Pregnancy is a vulnerable period for immune suppression in women and they experience various hormonal changes, which may lead to a greater chance of being infected with HPV (10). It is reported that the infection rate of High-risk HPV among pregnant women is 82%, while that among non-pregnant women is 10.4% (27). In this study, persistent High-risk HPV abnormalities were not associated with the progression of CIN and might be related to the fact that High-risk HPV has not yet turned negative after childbirth. During pregnancy, even if the pregnancy itself is not risky, the cervix may undergo changes. For instance, alterations in the shape or size of the cervix, changes in squamous and glandular epithelial cells, and increased blood vessels can affect cervical cytology and colposcopy (28). Therefore, for suspected cases, it is recommended to recheck cervical cytology and colposcopy during pregnancy. If necessary, a cervical tissue biopsy should be performed. At present, there is no reliable research on whether pregnancy affects cervical pathology, so pathological diagnosis remains the gold standard.

This study was conducted on pregnant women. The research subjects were pregnant women who were required to undergo colposcopy during pregnancy, so the clinical sample size was small. In particular, the sample size of the pregnancy biopsy group in this study was only 34 cases and 27 patients did not undergo postpartum colposcopy. This may limit statistical power. This is the limitation of this study. Previous studies also have the problem of having less data on the research subjects, and there are no updated data studies (29). In this study, only the first reexamination results within one year after giving birth were followed up, and no long-term follow-up was conducted. Therefore, this study has certain limitations. More data and multi-center studies need to be collected in the future to make the data more persuasive. This is also the future research direction of this study.

## Conclusion

Our study demonstrates that conservative management is appropriate for high-grade CIN during pregnancy when invasive carcinoma has been excluded through thorough prenatal evaluation. In such cases, we recommend regular surveillance with cervical cytology and HPV testing. Importantly, colposcopy alone provides substantial clinical value for pregnancy monitoring, even when patients decline cervical biopsy.

Key findings reveal that persistent cytological abnormalities strongly correlate with CIN prognosis in pregnant women, while persistent high-risk HPV infection shows no significant association with disease outcomes. These results underscore the particular importance of cytological monitoring in the management of CIN during pregnancy.

## Data Availability

The original contributions presented in the study are included in the article/supplementary material, further inquiries can be directed to the corresponding author.
